# Global transcriptome profiling identifies *KLF15* and *SLC25A10* as modifiers of adipocytes insulin sensitivity in obese women

**DOI:** 10.1371/journal.pone.0178485

**Published:** 2017-06-01

**Authors:** Agné Kulyté, Anna Ehrlund, Peter Arner, Ingrid Dahlman

**Affiliations:** Lipid laboratory, Department of Medicine H7, Karolinska Institutet, Stockholm, Sweden; University of Minnesota Twin Cities, UNITED STATES

## Abstract

Although the mechanisms linking obesity to insulin resistance (IR) and type 2 diabetes (T2D) are not entirely understood, it is likely that alterations of adipose tissue function are involved. The aim of this study was to identify new genes controlling insulin sensitivity in adipocytes from obese women with either insulin resistant (OIR) or sensitive (OIS) adipocytes. Insulin sensitivity was first determined by measuring lipogenesis in isolated adipocytes from abdominal subcutaneous white adipose tissue (WAT) in a large observational study. Lipogenesis was measured under conditions where glucose transport was the rate limiting step and reflects *in vivo* insulin sensitivity. We then performed microarray-based transcriptome profiling on subcutaneous WAT specimen from a subgroup of 9 lean, 21 OIS and 18 obese OIR women. We could identify 432 genes that were differentially expressed between the OIR and OIS group (FDR ≤5%). These genes are enriched in pathways related to glucose and amino acid metabolism, cellular respiration, and insulin signaling, and include genes such as *SLC2A4*, *AKT2*, as well as genes coding for enzymes in the mitochondria respiratory chain. Two IR-associated genes, *KLF15* encoding a transcription factor and *SLC25A10* encoding a dicarboxylate carrier, were selected for functional evaluation in adipocytes differentiated *in vitro*. Knockdown of *KLF15* and *SLC25A10* using siRNA inhibited insulin-stimulated lipogenesis in adipocytes. Transcriptome profiling of siRNA-treated cells suggested that *KLF15* might control insulin sensitivity by influencing expression of *PPARG*, *PXMP2*, *AQP7*, *LPL* and genes in the mitochondrial respiratory chain. Knockdown of *SLC25A10* had only modest impact on the transcriptome, suggesting that it might directly influence insulin sensitivity in adipocytes independently of transcription due to its important role in fatty acid synthesis. In summary, this study identifies novel genes associated with insulin sensitivity in adipocytes in women independently of obesity. *KFL15* and *SLC25A10* are inhibitors of insulin-stimulated lipogenesis under conditions when glucose transport is the rate limiting step.

## Introduction

Insulin resistance (IR) is a key pathogenic factor behind type 2 diabetes (T2D) that often accompanies obesity [1]. Although the mechanisms linking obesity to T2D are not well understood, it is likely that alterations of adipose tissue function are involved [2]. Adipocytes have multiple functions including energy storage/supply, and generating endocrine signals. Expansion of white adipose tissue (WAT) mass leads to increased adipocyte size, accelerated mobilization of fatty acids, increased local inflammation and altered release of adipocyte hormones (adipokines), which may contribute to overall IR [1–3].

However, not all obese are IR as reviewed [4–6]. In a large pan European study insulin sensitivity was, on average, 15–25% lower in obese than in lean people and only 25% of the obese had a marked IR when it was defined as the 10th lowest percentile of insulin sensitivity in lean subjects [7]. There is a genetic predisposition to systemic IR, but otherwise the causes are poorly understood [8]. A previous study, applying global transcriptome profiling on subcutaneous WAT from nondiabetic individuals in the fasted state, reported association between systemic insulin sensitivity and expression of genes related to lipid metabolism and inflammation [9]. Furthermore, we recently investigated gene expression in abdominal subcutaneous WAT of non-obese and obese women in relation to systemic *in vivo* insulin sensitivity assessed after two hours hyperinsulinemic-euglycemic clamp [10].Surprisingly, systemic insulin resistance had a limited impact on the transcriptional response to insulin infusion and the difference between the insulin resistant and sensitive groups was much smaller than the overall effect of obesity *per se* on gene expression in WAT. Only about 100 transcribed genes responded differently to hyperinsulinemia in women with systemic insulin resistance compared to insulin sensitive women.

The aforementioned studies addressed systemic insulin resistance but did not directly investigate whether the adipocytes were sensitive or resistant to insulin. To address this we determined insulin sensitivity of isolated adipocytes from abdominal subcutaneous WAT in a large cohort [11]. There are many ways of measuring insulin sensitivity in adipocytes, such as inhibition of lipolysis and effects on glucose metabolism (oxidation, glucose transport, lipogenesis). Insulin-stimulated glucose transport correlates with insulin sensitivity *in vivo* as measured with euglycemic, hyperglycemic clamp [12]. However, direct assays of human fat cell glucose transport require large amounts of tissue and are therefore not suitable for clinical studies. For that reason we use a simple and less tissue consuming method: lipogenesis in isolated fat cells [11]. Lipogenesis is measured as incorporation of ^3^H-labeled glucose into fat cell lipids. At low glucose concentrations in the incubation, which is used herein, glucose transport is the rate limiting step. This means that the lipogenesis measurements actually reflect the effects of insulin on glucose transport, in a manner similar to the ““gold standard” 3-0-methyl glucose transport assay[11].

In order to identify adipose expressed genes controlling adipocyte insulin sensitivity, we performed global transcriptome profiling on collected WAT specimen in a subset of 9 lean, 21 obese with insulin resistant adipocytes (OIR) and 18 obese with insulin sensitive adipocytes (OIS); all were women. These investigations identified *SLC25A10* and *KLF15* as modulators of adipocyte insulin sensitivity in humans, the latter possibly by influencing the expression of several known regulators of insulin action on glucose and lipid metabolism.

## Materials and methods

### Patients

The subjects were selected from an ongoing study on the genetics of insulin-stimulated lipogenesis in isolated human subcutaneous adipocytes [13]. The subjects were recruited by local advertising and all lived in Stockholm, Sweden. Patients were examined at the Karolinska University Hospital in Huddinge. For the present investigation 419 subjects were assessed [body mass index (BMI) range 18–62 kg/m^2^ and age 20–64 years] between years 1995 to 2008. To define insulin sensitivity we used insulin-stimulated minus basal lipogenesis in isolated adipocytes (see below) for all healthy lean subjects (n = 71; BMI <25 kg/m^2^). We adopted the method of Ferrannini et al. using lean healthy subjects to define insulin resistance in order to avoid the influence of confounding factors such as obesity and T2D that could by themselves influence insulin action [7]. Values above the threshold for upper 10^th^ percentile were defined as high insulin sensitivity and values below the threshold for lower 10^th^ percentile were defined as low sensitivity. For this study, we finally selected women from which we had stored frozen abdominal subcutaneous WAT samples and who were either lean (n = 9) or obese (BMI ≥30 kg/m^2^). The obese were categorized into either high (OIS, n = 21) or low (OIR, n = 18) insulin sensitivity based on insulin-stimulated minus basal lipogenesis in isolated adipocytes according to the definition above. Clinical characteristic are shown in Table 1, including information about medications.

**Table 1:** Clinical characteristics.

	Lean (n = 9)		OIS (n = 21)		OIR (n = 18)		*P*
	Average	SD	Average	SD	Average	SD	OIS-OIR
Age, years	37	11	36	8	40	8	0.14
BMI, kg/m^2^	23	1	41	5	40	6	0.4
Waist circumference, cm	80	4	121	11	124	11	0.29
Diastolic blood pressure (mm Hg)	75	9	75	8	83	9	0.01
Systolic blood pressure (mm Hg)	121	20	122	13	134	18	0.028
Plasma glucose, mmol/l	4.7	0.5	5.2	0.6	5.3	0.4	0.54
Plasma insulin, mU/l	6	4.3	11.4	4.6	16.7	9.7	0.017
HOMA_IR_	1.3	1.1	2.6	1.1	4.0	2.4	0.016
Plasma cholesterol, mmol/l	4.4	0.7	4.6	0.7	5.1	0.9	0.069
Plasma HDL cholesterol, mmol/l	1.6	0.3	1.3	0.3	1.1	0.2	0.03
Plasma triacylglycerides, mmol/l	0.8	0.3	1.4	0.7	1.7	0.7	0.13
Adipocyte volume, picolitres	424	174	864	167	949	124	0.13
Basal lipogenesis, nmol/10^7^cells	2.9	2.5	2.8	2.2	0.5	0.3	2.9x10^-8^
Insulin stimulated lipogenesis, nmol/10^7^cells	6.0	4.7	7.1	4.8	0.8	0.4	2.9x10^-12^
Insulin-basal lipogenesis, nmol/10^7^ cells	3.0	2.3	4.3	3	0.3	0.2	2.2x10^-13^
Insulin pD_2_ of lipogenesis	14.4	1.6	13.7	1.6	11.8	2.4	0.013

Values are mean+SD. OIR and OIS groups were compared with Student’s t-test. Blood pressure, plasma insulin, HOMA_IR_, HDL, triacylglycerides, and lipogenesis values were Log10 transformed before analysis to become normally distributed. BMI, waist circumference, adipocyte volume and pD_2_ deviated from normality and were analyzed by Kruskal-Wallis test.

One lean woman took contraceptive pills. Two OIS women took contraceptive pills, and one women a selective serotonin reuptake inhibitor (SSRI). Three OIR women took SSRI and one woman another antidepressant drug (Duloxetine). Two OIR women were prescribed a combination of thiazide and amiloride diuretics. Finally, the following pharmaceuticals were each prescribed to one OIR woman: angioten converting enzyme inhibitor, statin, contraceptive pills, Infliximab (TNFA receptor antagonist), Methotrexate, non-steroid anti-inflammatory drug, selective histamine H1-receptor antagonist, thyroid hormone (T4), the antieleptic drug topiramate, the antipsychotic drug Ziprasidone, the opiod receptor modulator Buprenorphine, and an inhalable corticosteroid and inhalable selective beta-2 stimulatory agonist.

For functional studies, subcutaneous WAT was obtained from healthy subjects undergoing cosmetic liposuction. In this group, there was no selection for age or sex. In a subgroup of these tissue donors (n = 12), global transcriptome profiles were determined during *in vitro* differentiation of primary human precursor cells into adipocytes as has been reported previously [14]. The Regional board of ethics in Stockholm approved the study, and written informed consent was obtained from all participants. All clinical investigation have been conducted according to the principles expressed in the Declaration of Helsinki.

### Clinical examination

Patients came to the laboratory for clinical examination following an overnight fast. A nurse determined height, weight, and waist circumference. A venous blood sample was obtained for measuring lipids and glucose at the routine clinical chemistry laboratory. Serum insulin was measured by ELISA (Mercodia, Uppsala, Sweden). Systemic insulin resistance was estimated according to the Homeostasis model adjustment (HOMA_IR_) formula [15]. Following the clinical examination an abdominal subcutaneous WAT biopsy was obtained by needle aspiration, as described [16]. Tissue pieces were rapidly rinsed in saline, and a small part was immediately used for quantification of adipocyte volume and lipogenesis. The rest was frozen in liquid nitrogen and kept at -70°C.

### Cell isolation from subcutaneous WAT biopsies

Mature adipocytes from biopsies were prepared using the collagenase procedure as described [17]. Mean adipocyte weight and volume were determined as described [18, 19].

### Primary adipocyte cultures

Primary adipocyte culture for *in vitro* studies was obtained from subcutaneous WAT from healthy non-obese men and women (BMI ≤30 kg/m^2^) undergoing cosmetic liposuction. The stroma vascular fraction (SVF) cells were isolated as described [20]. The precursor cells obtained from separate individuals were not mixed. Part of plastic-adherent SVF cells were directly plated (30,000–50,000 cells/cm^2^) and differentiated to adipocytes as described [20]. The degree of differentiation was controlled under the microscope as accumulation of lipids, and response to insulin in lipogenesis assay was evaluated (see below). Cultures with a differentiation degree below ~80% were discarded. Remaining SVF cells were suspended in fetal calf serum (FCS) supplemented with 10% DMSO and stored in liquid nitrogen until further usage. Upon the experiments, the SVF was thawed, washed with inoculation medium, plated and differentiated as described above.

### Insulin-stimulated lipogenesis in *ex vivo* isolated mature adipocytes

Lipogenesis in mature adipocytes isolated from WAT biopsies by collagenase procedure was conducted as described [19]. In brief, isolated adipocytes were incubated in an albumin containing buffer with (^3^H) glucose at a final glucose concentration of 1 μmol/l at which glucose transport is the rate limiting step. After 2 h of incubation at 37°C adipocyte lipids were extracted and counted for radioactivity. Data were expressed as amount of glucose incorporated into lipids (nmol glucose x 2 h^−1^ x [10^7^ adipocytes]^−1^). The basal lipogenesis (no insulin present) and lipogenesis at the maximum effective concentration for insulin were measured. The sensitivity of the adipocytes to insulin was expressed using equation pD_2_ = -log(EC_50_), where EC_50_ is the concentration (mol/l) of the hormone that produces a half-maximum effect, and was calculated from logistic conversion of the concentration-response curve as described [19]. In methodological experiments we compared insulin stimulated lipogenesis with *in vivo* insulin-induced glucose disposal rate during euglycemic hyperinsulinemic clamp, the latter was determined as described [10]. In 129 subjects, who in underwent both examinations, the results were positively correlated (r = 0.4; *P* <0.0001).

### Insulin-stimulated lipogenesis in *in vitro* differentiated adipocytes

To measure lipogenesis in SVF-derived adipocytes differentiated *in vitro*, the cells were first washed once with DMEM without glucose (Biochrom AG, Berlin, Germany) and incubated in insulin-free DMEM (Biochrom AG) supplemented with 1 μM glucose for three hours. Following the starvation, the cells were incubated for two hours in the presence or absence of 10^−7^ mol/l insulin and D-[3-^3^H] glucose (37 MBq/ml; Perkin Elmer-Cetus, Norwalk, CT, USA) diluted 1:1000. Subsequently, the cells were washed three times with cold PBS and lysed in 0.1% SDS/H_2_O. Ten μl of the lysate was saved for determination of protein concentration using Pierce BCA Protein determination kit (Thermo Fisher Scientific, Lafayette, CO). The rest of the lysate was transferred to cuvettes containing scintillation fluid (toluene with 5 g/l 2.5-Diphenyloxazol and 0.3 g/l 1.4-Bis (4-methyl-5-phenyl-2-oxazolyl)-benzene; Sigma-Aldrich, St. Louis, MO) and CPM was recorded after overnight phase separation.

### Transfection of siRNA

*In vitro* differentiated adipocytes (day 10–12 post induction) were transfected with ON-TARGETplus SMARTpool small interfering RNAs (siRNAs) targeting *KLF15*, *SLC25A10* and *SLC2A4* or non-targeting siRNA pool at 40 nM final concentration (Dharmacon, Lafayette, CO) in 24- or 48-well plates and HiPerfect Transfection Reagent (Qiagen), respectively 4.5 or 2 μl according to the manufacturer’s protocol. The cells were incubated for 48 or 72 hours at which time *in vitro* lipogenesis was assessed in cells plated on 48-well plates and RNA were collected.

### RNA isolation, cDNA synthesis and real-time PCR

Total RNA was extracted from WAT specimens (300 mg) using RNeasy Lipid tissue (Qiagen, Hilden, Germany) according to the manufacturer’s recommendations. Total RNA from samples obtained in *in vitro* experiments was extracted using NucleoSpin RNA II kit (Macherey-Nagel, Düren, Germany) according to the manufacturer’s instructions. RNA concentration as well as purity was measured using a Nanodrop ND-1000 Spectrophotometer (Thermo Fisher Scientific) and high quality RNA was confirmed using the Agilent 2100 Bioanalyzer (Agilent Technologies, Palo Alto, CA, USA). Reverse transcription was performed using the iScript cDNA synthesis kit (Qiagen) and random hexamer primers (Invitrogen, Carlsbad, CA). Quantitative real-time PCR was performed using commercial TaqMan probes (Applied Biosystems). Gene expression was normalized to the internal reference gene *LRP10*. Relative expression was calculated using the 2[-Delta Delta C(T)] method [21].

### Microarray assay

Gene 1.1 ST Arrays were used to profile gene expression in WAT from clinical samples. In a number of previous studies we have shown that by global transcriptome profiling on WAT biopsies it is possible to identify adipocyte expressed genes of clinical relevance. Furthermore, transcriptome profiling on WAT pieces avoids the impact on gene expression caused by the protocol for isolating adipocytes. From high-quality total RNA we prepared and hybridized biotinylated complementary RNA to the arrays, and then washed, stained and scanned the slides using standardized protocols (Affymetrix Inc., Santa Clara, CA, USA). The arrays were subsequently pre-processed using Affymetrix Expression Console with the following settings; summarization: PLIER, and normalization: global-median. To allow comparisons of transcript levels between samples, all samples were subjected to an all-probeset scaling-to-target signal of 250. The Gene 1.1 ST Arrays measure the expression of 28,869 transcripts. We filtered for probesets linked to a gene symbol, and, second, probesets with average signal among all samples >50, leaving 13,798 probesets for phenotypic analysis. The threshold >50 was chosen since it excluded transcripts that should not be present in WAT. Expression levels between the obese OIR and OIS groups were compared with Significance analysis of arrays (SAM) applying 1,000 permutations applying a false discovery rate of (FDR) <5% [22]. For genes represented with more than one probeset on the array, we show results for the probeset with highest average signal.

Gene 2.1 ST arrays were used for global transcriptome profiling of primary adipocyte cultures treated with siRNA against *KLF15* (n = 8), *SLC25A10* (n = 8), or non-targeting siRNA control (n = 8). These arrays were pre-processed with RMA, which includes normalization, summarization of probes to probesets, and background correction. We used RMA since phenotypic analysis was performed in Limma, which requires Log-based data [23]. One RNA sample was labelled and hybridized twice to the microarrays; both experiments produced similar expression profiles. For this sample we used the average of the expression values according to RMA in subsequent analysis. We filtered for probesets linked to a gene symbol but excluded probesets linked to non-coding RNAs. Next we excluded ~9,000 probesets (9,012 in *KLF15* and 9,019 in *SLC25A10* analysis) with average expression signal ≤8, and subsequently ~2,340 probesets (2,345 in *KLF15* and 2,338 in *SLC25A10* analysis) with lowest SD/average expression based on analysis of all samples leaving 11,358 probesets for subsequent phenotypic analysis. Limma software was used to assess the impact of gene knockdown on the transcriptome using a model that takes into account number of subjects, duplicate cell culture samples, and treatment [23]. False discovery rat (FDR) <10% was used as cutoff to identify *KLF15* and *SLC25A10* target genes; the less stringent threshold was chosen due to small sample size.

Webgestalt was used to identify gene sets that were over-represented among differentially expressed as compared to all analyzed genes [24]. Microarray data have been submitted to GEO with accession number GSE94753.

### Statistical analyses

Statistical analyses were performed in JMP v. 12. Shapiro-Wilks test was used to test whether the distribution of clinical variables deviated from normality. Two-group comparisons of normally distributed variables were performed by two-sided Student’s t-test and otherwise by Kruskal-Wallis test. Quantitative relationships were evaluated by least square regression.

## Results

### Clinical characteristics

Clinical characteristics of the studied cohort are shown in Table 1. The OIR group was defined by insulin-stimulated minus basal lipogenesis in isolated adipocytes below the threshold for lower 10^th^ percentile for all healthy lean subjects, and the OIS group by insulin-stimulated minus basal lipogenesis above the threshold for upper 10^th^ percentile. The two obese groups were matched for BMI, waist circumference and adipocyte size; all of which may influence insulin sensitivity independently of insulin-stimulated lipogenesis. All groups were matched for age. The average age was 38 years (range 22–60 years). The OIR women displayed markedly lower systemic insulin sensitivity as estimated by HOMA_IR_ than the OIS women.

### Genes associated with adipose insulin resistance

462 probe sets representing 432 genes were differentially expressed between the OIR and OIS group (FDR ≤5%) (S1 Table). According to the Webgestalt tool, the 432 insulin resistance-associated genes were enriched in pathways related to glucose and amino acid metabolism, and cellular respiration, as compared to all analyzed genes (selected pathways are listed in Table 2; all significant pathways a listed in S2 Table). The Insulin signaling pathway had adjusted *P* = 0.06. Genes involved in glucose uptake and insulin signaling were with one exception (*NUP93*) expressed at lower levels in the OIR group, and included *SLC2A4*, *AKT2*, as well as genes coding for several enzymes in the mitochondria respiratory chain (Table 3). The expression pattern of these genes with known function was consistent with the difference in sensitivity to insulin between groups.

**Table 2:** Selected pathways over-represented among differentially expressed genes between OIR and OIS women^a^.

Pathway	differentially expressed genes		
	observed	expected	adjusted *P*
**Pathway Commons**			
Oxidative decarboxylation of pyruvate to acetyl CoA by pyruvate dehydrogenase	23	2.16	1.74x10^-15^
Regulation of pyruvate dehydrogenase complex	23	2.18	1.74x10^-15^
Oxidative decarboxylation of alpha-ketoadipate to glutaryl CoA by alpha-ketoglutarate dehydrogenase	22	2.12	8.06x10^-15^
Oxidative decarboxylation of alpha-ketoglutarate to succinyl CoA by alpha-ketoglutarate dehydrogenase	21	2.07	1.09x10^-14^
Isoleucine catabolism	22	2.29	1.09x10^-14^
Glucose uptake	29	4.47	1.09x10^-14^
Glucose 6-phosphate is isomerized to form fructose-6-phosphate	27	3.77	1.09x10^-14^
Regulation of Insulin Secretion	29	4.49	1.09x10^-14^
Pyruvate metabolism	26	3.49	1.09x10^-14^
Insulin effects increased synthesis of Xylulose-5-Phosphate	27	3.73	1.09x10^-14^
**Wikipathways**			
Electron Transport Chain	17	1.92	7.94x10^-11^
Oxidative phosphorylation	12	1.11	4.72x10^-09^
TCA Cycle	6	0.61	0.0001
Eukaryotic Transcription Initiation	6	0.87	0.0006
AMPK signaling	6	1.37	0.0046
Androgen Receptor Signaling Pathway	8	2.31	0.0046
TNF-alpha/NF-kB Signaling Pathway	11	3.95	0.0046
estrogen signalling	6	1.53	0.0068
mRNA processing	8	2.64	0.0074
Insulin Signaling	7	3.27	0.0603

a. Webgestalt was used to determine whether genes differentially expressed between OIR and OIS women were over-represented for specific gene sets as compared to all analyzed genes. Only gene sets with at least five differentially expressed genes were considered. Otherwise default settings were used i.e. statistics test: hypergeometric; correction for multiple testing: Benjamini-Hochberg. Separate runs were used for analysis of the different gene sets databases.

**Table 3:** Glucose uptake and insulin signaling genes differentially expressed between OIR and OIS women.

Gene	OIS (n = 21)	OIR vs OIS
	average^a^	ratio
**Glucose uptake**		
*NDUFS2*	470	0.91
*NDUFB8*	726	0.92
*PDHX*	622	0.89
*COX8A*	863	0.90
*NDUFV1*	574	0.91
*HMBS*	303	0.84
*NDUFC2*	1783	0.94
*ACACB*	2201	0.87
*NUP93*	255	1.14
*COX4I1*	1398	0.93
*SLC2A4*	252	0.75
*ATP5G1*	1274	0.90
*SLC25A10*	243	0.79
*SDHC*	1928	0.91
*SLC25A11*	618	0.89
*ATP5D*	346	0.89
*GCDH*	154	0.85
*NDUFA3*	727	0.86
*COX5B*	1878	0.88
*SUCLG1*	859	0.86
*NDUFV3*	660	0.87
*ATP5J*	529	0.90
*NDUFA6*	1119	0.92
*NDUFS4*	399	0.89
*NDUFA2*	407	0.87
*MDH2*	1350	0.91
*PDHA1*	718	0.85
*SLC25A6*	3486	0.93
*IDH3G*	972	0.94
*SLC25A6*	3486	0.93
**Insulin signaling**		
*PTPRF*	855	0.91
*TBC1D4*	416	0.88
*SLC2A4*	252	0.75
*VAMP2*	569	0.91
*AKT2*	1371	0.89
*GYS1*	471	0.80
*SRF*	375	0.89

a. Average expression signal of specific genes on the arrays in the OIS group.

**Fig 1:**
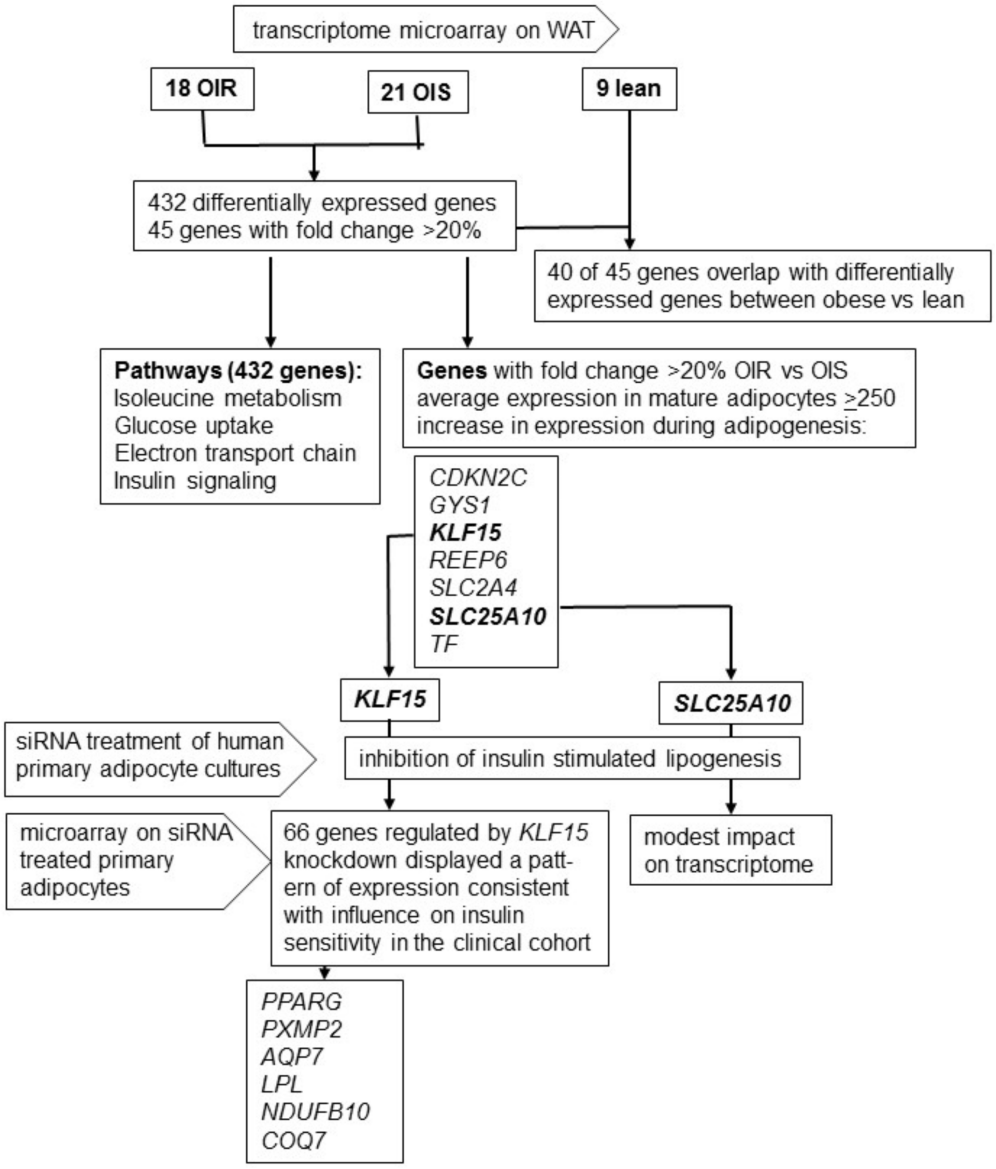
Flowchart describing the project.

In general fold change in expression between groups was small. Only nine genes displayed fold change in expression >30% whereas no gene displayed fold change >50% between OIR and OIS women. Among the 45 genes with fold change in expression ≥20% between the OIR and OIS groups, 40 genes displayed directionally consistent, and in most cases nominally significant, difference in expression between all obese versus lean women (Table 4). Among genes expressed at lower levels in the OIR group, some have been reported to be involved in insulin signalling and/or adipogenesis-regulating pathways, i.e. *SLC2A4* encoding GLUT4, *AXIN2* [25, 26], *TF* encoding Transferrin *[27]*, *KLF15* [28], and *CDKN2C [29].* Five genes did not display directionally consistent expression pattern in the OIR versus OIS, and obese versus lean comparisons (*AXIN2*, *DLEC1*, *ANKRD36B*, *LOC441666*, *CAB39L*); these genes are besides *AXIN2* poorly defined as regards function. All five are expressed at low levels in adipocytes, and none display increased expression during adipogenesis (FDR ≤1%) (Table 4). We did therefore not study these genes in more detail. A flowchart describing the project is shown in Fig 1.

**Table 4:** Genes differentially expressed between OIR and OIS women with FDR ≤5% and fold change in expression 20%.

	Subcutaneous WAT						Primary adipocyte cultures			
	Lean (n = 9)	OIS (n = 21)	OIR (n = 18)	OIR vs OIS	Obese vs lean		day 4	day 12	day 4 vs 12	
Gene	Average			Ratio	ratio	*P*^a^	Average		*P*^a^	
*COL6A6*	175	80	42	0.52	0.36	2.90x10^-06^	66	141	7.97x10^-03^	
*SCUBE1*	130	80	41	0.52	0.48	7.52x10^-04^	77	69	1.77x10^-01^	
*TF*	1181	816	558	0.68	0.59	2.41x10^-04^	53	7373	5.01x10^-04^	^b^
*REEP6*	207	211	146	0.69	0.88	3.68x10^-01^	42	252	5.28x10^-04^	^b^
*KLF15*	340	228	165	0.72	0.59	2.96x10^-06^	141	267	5.30x10^-03^	^b^
*AXIN2*	84	122	90	0.74	1.27	7.14x10^-02^	58	52	2.39x10^-01^	
*SLC7A10*	820	475	351	0.74	0.51	2.47x10^-07^	523	566	2.48x10^-01^	
*CDKN2C*	286	209	156	0.75	0.65	6.43x10^-05^	256	841	8.41x10^-04^	^b^
*SLC2A4*	471	252	188	0.75	0.47	9.77x10^-11^	16	387	8.80x10^-05^	^b^
*HRSP12*	204	177	134	0.76	0.77	4.57x10^-04^	226	309	6.08x10^-02^	
*CTNNAL1*	318	237	180	0.76	0.66	7.37x10^-06^	252	375	8.00x10^-02^	
*DLEC1*	95	125	97	0.77	1.18	1.24x10^-01^	89	102	2.41x10^-01^	
*ITPKC*	131	140	109	0.78	0.96	7.24x10^-01^	94	80	2.18x10^-01^	
*FGFRL1*	504	338	266	0.78	0.61	2.67x10^-10^	120	160	5.68x10^-02^	
*GPT*	134	94	74	0.79	0.63	2.60x10^-06^	42	73	9.66x10^-04^	^b^
*GLIS2*	163	168	132	0.79	0.93	4.55x10^-01^	156	210	3.32x10^-02^	
*SPON1*	1183	933	733	0.79	0.71	6.21x10^-06^	1058	334	2.78x10^-05^	^b^
*SLC25A10*	270	243	191	0.79	0.81	2.99x10^-02^	73	308	6.49x10^-05^	^b^
*TBC1D24*	255	220	175	0.80	0.78	3.20x10^-03^	97	114	1.33x10^-01^	
*LDHD*	254	162	130	0.80	0.58	5.79x10^-08^	61	318	3.83x10^-04^	^b^
*PER1*	529	481	387	0.80	0.83	4.98x10^-02^	62	55	2.50x10^-01^	
*GYS1*	513	471	379	0.80	0.84	3.21x10^-02^	261	558	1.15x10^-03^	^b^
*RPS27L*	202	236	283	1.20	1.28	1.07x10^-03^	385	576	3.61x10^-02^	
*SLC4A7*	115	142	170	1.20	1.35	1.68x10^-04^	342	247	8.98x10^-03^	^b^
*SEL1L3*	136	165	198	1.20	1.33	4.80x10^-04^	795	215	5.41x10^-05^	^b^
*OBFC1*	111	105	127	1.21	1.04	4.69x10^-01^	136	252	8.25x10^-03^	
*ANKRD44*	180	210	253	1.21	1.28	2.38x10^-03^	53	63	1.64x10^-01^	
*NAAA*	173	198	241	1.22	1.26	5.23x10^-03^	146	128	1.53x10^-01^	
*SNRPD3*	221	219	268	1.22	1.10	2.02x10^-01^	1161	1306	2.89x10^-01^	
*ANKRD36B*	635	552	685	1.24	0.97	5.94x10^-01^	178	102	4.04x10^-05^	^b^
*HMCN1*	259	250	310	1.24	1.07	3.89x10^-01^	139	107	1.83x10^-01^	
*HERC2P4*	475	558	694	1.24	1.31	3.83x10^-03^	354	359	9.05x10^-01^	
*LOC441666*	375	324	405	1.25	0.96	6.95x10^-01^	208	243	1.57x10^-01^	
*SCPEP1*	318	396	496	1.25	1.39	2.95x10^-03^	1260	904	2.90x10^-03^	^b^
*HSD17B11*	546	559	701	1.25	1.14	6.76x10^-02^	409	266	3.00x10^-03^	^b^
*CFH*	665	937	1187	1.27	1.58	1.07x10^-04^	689	850	1.23x10^-01^	
*CAB39L*	258	194	248	1.27	0.85	7.24x10^-02^	113	153	3.33x10^-02^	
*FN1*	1336	1825	2328	1.28	1.54	8.54x10^-04^	2710	1757	6.38x10^-03^	^b^
*FAM96A*	147	157	200	1.28	1.20	2.65x10^-02^	624	483	1.96x10^-02^	
*NOX4*	115	181	232	1.28	1.78	1.30x10^-05^	139	99	9.97x10^-02^	
*CFHR1*	184	258	346	1.34	1.62	2.75x10^-04^	177	183	8.18x10^-01^	
*CFHR2*	50	74	100	1.36	1.71	1.98x10^-04^	42	48	4.54x10^-01^	
*VCAN*	307	489	679	1.39	1.88	8.70x10^-04^	1732	507	2.00x10^-03^	^b^
*CTSH*	291	346	489	1.41	1.41	1.86x10^-02^	364	417	4.64x10^-01^	
*VNN1*	25	56	82	1.45	2.71	3.14x10^-06^	52	48	4.08x10^-01^	

a. Gene expression in obese and lean groups were compared with Student's t-test.

b. Significantly different expression day 4 vs 12 according to SAM with FDR ≤1%.

As we could not study all differentially expressed genes functionally due to limited resources we narrowed down the list by filtering on the following criteria: genes with fold change ≥20% between the OIR and OIS group, average expression signal in mature *in vitro* differentiated adipocytes ≥250 arbitrary units, and with increased expression during adipogenesis (FDR ≤1%) [14]. Seven genes fulfilled these criteria, *TF*, *REEP6*, *KLF15*, *CDKN2C*, *GYS1*, *SLC2A4*, and *SLC25A10* (Table 2). Based on initial screening experiments and reported gene functions, we considered *KLF15* and *SLC25A10* as the most likely involved in development of adipocyte IR. *KLF15*, which encodes a transcription factor, was selected because it has been reported to stimulate adipogenesis and target *SLC2A4* in rodent models [28]. *SLC25A10*, a dicarboxylate transporter, has been reported to play an important role in fatty acid synthesis and hereby to control lipid accumulation in adipocytes [30].

**Fig 2:**
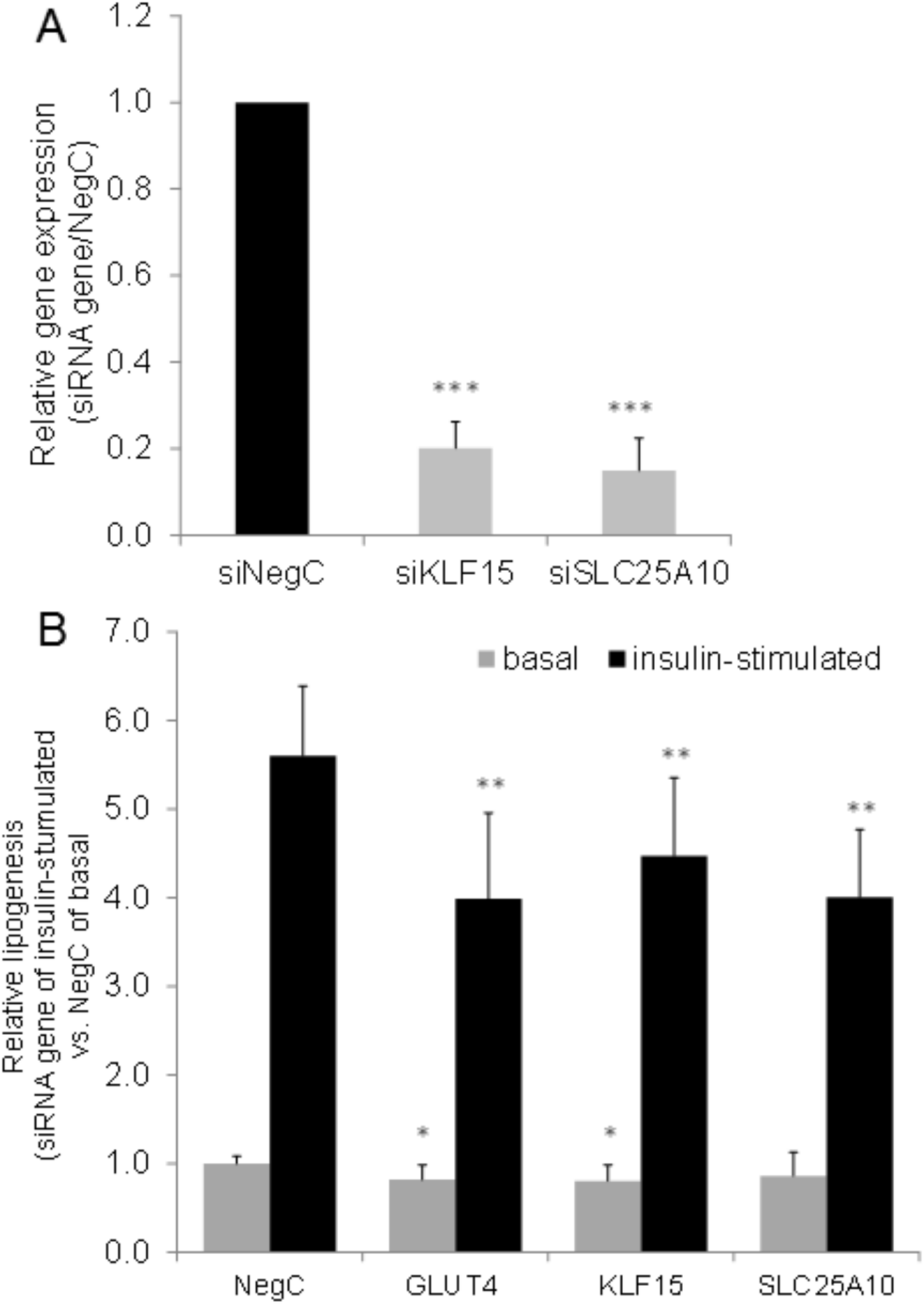
Effects of *KLF15* and *SLC25A10* knockdownon lipogenesis *in vitro*. **A.** *KLF15* and *SLC25A10* were knocked down using 40nM of siRNA in SVF-derived human adipocytes differentiated *in vitro* and expression of the genes evaluated using real-time PCR. Results were analyzed using Students t-test and are presented as relative fold change ± SD vs. negative control. **B.** SVF-derived adipocytes differentiated *in vitro* were transfected with 40 nM of siRNA against *KLF15* and *SLC25A10* for 48 hours followed by evaluation of basal and insulin-stimulated lipogenesis. Relative insulin-stimulated lipogenesis was calculated against non-targeting siRNA NegC at insulin-stimulated state. Induction of lipogenesis by insulin for NegC was minimum 3-fold in all experiments. Results are based on three to five biological/independent experiments.*p<0.05, **p<0.01 and ***p<0.001.

siRNA treatment resulted in efficient knockdown of *KLF15* and *SLC25A10* mRNA (Fig 2A), as well as inhibited insulin stimulated lipogenesis (Fig 2B). These results are consistent with the lower expression of these genes in OIR compared to OIS women. Knockdown of *SLC2A4* was used as a positive control for the lipogenesis assay.

### *KLF15* and insulin resistance in human adipocytes

To delineate pathways controlled by KLF15 signaling in human adipocytes, we characterized the effects of *KLF15* knockdown on the global transcriptome. 366 genes were differentially expressed between *KLF15* knockdown and control cells with FDR 10% (S3 Table). Overall, 18 genes targeted by *KLF15* overlapped with genes differentially expressed between OIR and OIS women. 15 of 18 genes displayed a consistent pattern of expression in the *KLF15* siRNA treated cells and in the clinical cohort, e.g. downregulated by *KLF15* knockdown and lower expression in OIR women. These 15 genes include one gene in the insulin signaling (*GYS1)* and one in the glucose transport (*GCDH*) pathways. Besides *GYS1* and *GCDH*, only *PCYT2* of the 15 candidate genes, has previously been linked to relevant metabolic disease phenotypes [31]. However, contrary to expected, *PCYT2* knockdown in human adipocytes increased insulin-stimulated lipogenesis (results not shown). *KLF15* has been reported to control expression of glucose transporter *SLC2A4* in rodents *[32];* however *KLF15* knockdown did not affect *SLC2A4* in our human cells (results not shown).

**Fig 3:**
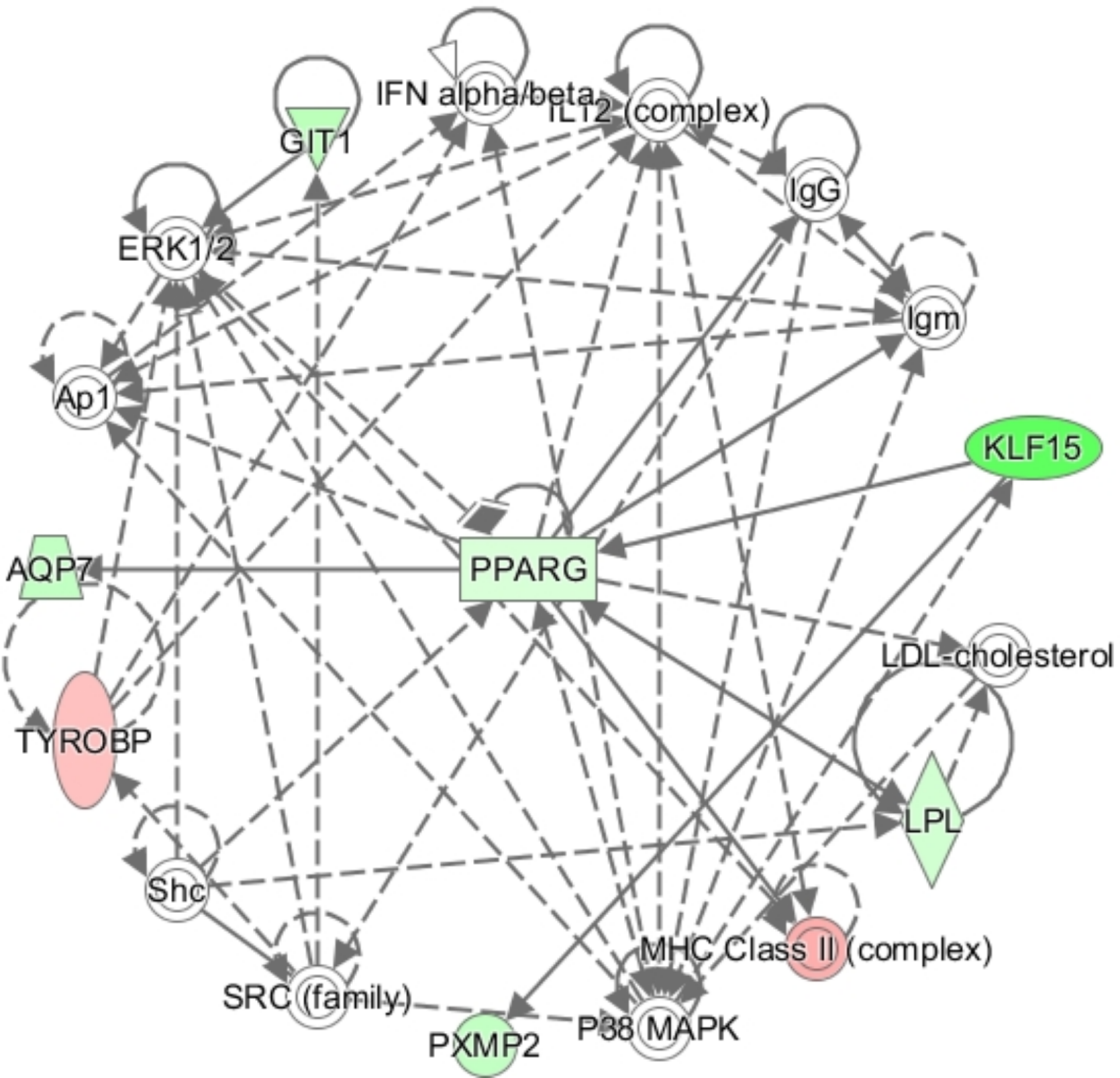
Ingenuity analysis of *KLF15* target genes associated with insulin stimulated lipogenesis *in vivo*.

In order to further elucidate molecular pathways linking *KLF15* to insulin sensitivity we regressed insulin stimulated lipogenesis in the obese subset of the clinical cohort on expression of the 366 *KLF15* target genes. Expression of 77 genes were nominally associated with insulin stimulated minus basal lipogenesis of which 66 genes displayed a pattern of expression consistent with regulation by *KLF15* and influence on insulin sensitivity in the clinical cohort (S4 Table). Interestingly, in analysis of gene sets over-represented among these 66 *KLF15*-target genes compared to all analyzed genes expressed in the cells, insulin resistance scored highest (adjusted *P* 9.7x10^-5^) (Table 5). Adipogenesis was the most strongly enriched Wikipathway (adjusted *P* 0.005). Ingenuity analysis of the 66 genes identified *PPARG* and *PXMP2* as targets of *KLF15*, and *AQP7* as well as *LPL* as targets of *PPARG* (Fig 3). Other *KLF15* target genes of potential importance for insulin sensitivity include the *NDUFB10* and *COQ7* genes encoding mitochondrial enzymes (S4 Table).

**Table 5:** Gene sets enriched among target genes in *KLF15* knockdown.

**Disease gene set**	**Insulin Resistance**
Number of genes; observed = 7; expected = 1; adjusted *P* = 9.7x10^-05^	
Gene	OIR vs OIS
*GYS1*	0.82
*LPL*	0.84
*AZGP1*	0.69
*PPARG*	0.86
*SEPP1*	1.16
*AQP7*	0.79
*ADRA2A*	0.69
**Wikipathway**	**Adipogenesis**
Number of genes; observed = 4; expected = 0.6; adjusted *P* = 0.005	
Gene	OIR vs OIS
*KLF15*	0.54
*LPL*	0.84
*CEBPA*	0.86
*PPARG*	0.86

### *SLC25A10* and insulin resistance

Furthermore, we also examined the effects of *SLC25A10* knockdown on the transcriptome in human adipocytes. 165 genes were differentially expressed between *SLC25A10* knockdown and control cells with FDR ≤10% (S5 Table). Three genes targeted by *SLC25A10* overlapped with genes differentially expressed between OIR and OIS women. Two of three genes displayed a pattern of expression consistent with regulation by *SLC25A10* and influence on insulin sensitivity in the clinical cohort, *ACVR1C* and *SLC41A1*. *SLC25A10* and *KLF15* did not target one another. One gene in the glucose transport and insulin signaling pathways was regulated by *SLC25A10* knockdown, *MAPK6*, but this gene was not differentially expressed in WAT of OIR and OIS women. In order to further elucidate molecular pathways linking *SLC25A10* to insulin sensitivity we regressed insulin stimulated lipogenesis in the obese subset of the clinical cohort on expression of the *SLC25A10* target genes. 27 genes were nominally associated with insulin stimulated minus basal lipogenesis of which 16 displayed a pattern of expression consistent with regulation by *SLC25A10* and influence on insulin sensitivity in the clinical cohort (S6 Table). The Webgestalt tool did not identify any over-represented pathways among these 16 genes associated with insulin sensitivity as compared to all analyzed genes. However, specific genes reported to be phenotypically linked to adipose functions and potentially involved in the regulation of insulin sensitivity include *ACVR1C*, which inhibits lipolysis [33], *PRKAR2A* which comprises a subunit of protein kinase A [34], *SEMA3E* which regulates adipose inflammation [35], and *SKP2 [36]* which controls adipocyte number.

## Discussion

The aim of this study was to define novel genes that may cause insulin resistance in human adipocytes independently of obesity. To assess insulin sensitivity we applied lipogenesis assay where glucose transport is the rate limiting step. By transcriptome profiling in a large human cohort including non-obese and obese with or without insulin resistance, combined with functional evaluation in adipocyte cultures of specific genes, this study identifies *KLF15* and *SLC25A10* as potential modulators of adipocyte insulin sensitivity. In addition, this study highlight potential roles for enzymes involved in glucose uptake and metabolism, and for genes controlling adipogenesis in development of adipocyte insulin resistance in obese women [37]. These findings shed new light on the complex signals linking obesity to development of insulin resistance.

In the study, siRNA knockdown of *KLF15* and *SLC25A10* resulted in a ~20% inhibition of insulin stimulated lipogenesis. The effect was modest in comparison with the more dramatic differences in insulin stimulated lipogenesis between OIR and OIS women in the clinical cohort. This effect is compatible with a model where insulin sensitivity displays a complex etiology; it is likely that several genes are involved and that different genes might be more or less important in different individuals. Thus, we consider *KLF15* and *SLC25A10* to be modulators on insulin sensitivity.

*KLF15* has previously been reported to be down-regulated in subcutaneous WAT and skeletal muscle from subjects with systemic insulin resistance [9], thus supporting an important role for *KLF15* not only in adipose tissue but in systemic insulin resistance. A number of target genes for *KLF15* including S*LC2A4* have been identified in rodent adipocyte [32, 38–41]. These genes, however, were not detected as targeted by *KLF15* in our experiments in human cells. The discrepancy could potentially be due to species specific effects of *KLF15*, due to the presence of different transcriptional cofactors in the different cell culture systems or due to limited sensitivity in our experiment leading to failure to detect all regulated genes. Importantly, the *KLF15* target gene *PPARG* reported in rodent models was confirmed in our human adipocytes [42]. In rodents, the *KLF15-PPARG* interaction signal is important for adipogenesis. However, *PPARG* is also a key factor controlling lipogenesis and insulin sensitivity in mature adipocytes [43]. Our siRNA knockdown of *KLF15* was performed late during *in vitro* differentiation of adipocytes, demonstrating that the *KLF15-PPARG* signal most likely controls adipocyte insulin sensitivity independently of adipogenesis. Whereas at least three other *KLFs* have been shown to bind to the *PPARG2* isoform promoter, binding sites of *KLF15* in the *PPARG* promoters have yet not been defined [44]. Thus, it is currently unknown whether the control of *PPARG* gene transcription by *KLF15* is direct, or indirect, by controlling the activity of other transcription factors which in turn control *PPARG* expression. Other transcription factors known to control *PPARG* gene expression [44, 45] were not among KLF15 target genes.

In the siRNA experiments we identified additional downstream targets of *KLF15* implicated in lipid storage including *LPL* involved in cellular uptake of free fatty acids [46], the glycerol channel *AQP7* involved in triglyceride synthesis [47], and *PXMP2* which is essential for mammary lipid homeostasis [48].AQP7 disruption elevates adipose glycerol kinase activity, accelerates triglyceride synthesis in adipocytesAQP7 disruption elevates adipose glycerol kinase activity, accelerates triglyceride synthesis in adipocytesAQP7 disruption elevates adipose glycerol kinase activity, accelerates triglyceride synthesis in adipocytes.

Knockdown of *SLC25A10* inhibited insulin stimulated lipogenesis with the same magnitude as *KLF15*. However, the transcriptional effects of *SLC25A10* knockdown were modest. A possible complementary explanation is that *SLC25A10* has direct effects on insulin stimulated lipogenesis. *SLC25A10* encodes a dicarboxylate carrier that transports e.g. malate and succinate across the mitochondrial inner membran dicarboxylate carrier dicarboxylate carrier edicarboxylate carrier dicarboxylate carrier. It has been reported to play an important role in fatty acid synthesis and hereby to control lipid accumulation in adipocytes [30] and knockdown could thus potentially directly impact lipogenesis without need for transcriptional alterations.

WAT from the OIR women was further characterized by lower expression of specific genes implicated in the regulation of adipogenesis, i.e. *TF [27]*, *AXIN2* [25], and *CDKN2C [29]*. This finding is in agreement with the established association between impaired adipogenesis and insulin resistance as reviewed [49]. Also, lower expression of genes in the mitochondria respiratory chain is consistent with reported role of mitochondrial dysfunction in insulin resistance and T2D [50, 51].

Finally, to assess the importance for whole body insulin resistance of the 432 genes whose expression associated adipocyte insulin resistance, we examined their expression in relevant published global transcriptome studies on subcutaneous WAT. Elbein et al. reported that the expression of 172 genes in subcutaneous WAT is associated with systemic insulin resistance [9]. Ten of these genes overlapped with genes associated with adipocyte insulin resistance in the present study; all displayed a directionally consistent change in expression (Table 6). Although the absolute number of genes overlapping between studies was small, possible due to limited sample size and power of both studies, these results highlight a potential role for dysregulated WAT gene expression in development of whole body insulin resistance. Nilsson et al. reported that expression of 197 genes in subcutaneous WAT is differentially expressed between twins discordant for T2D [52]. Six genes overlapped with the present study, of which four displayed consistent expression pattern (Table 6). The poor overlap with T2D may be due to limited importance of adipocyte insulin resistance for development of diabetes in this cohort, or the adipose transcriptome being dominated by changes that are secondary to hyperglycemia.

**Table 6:** Genes associated with adipocyte IR also associated with systemic insulin resistance or T2D.

	Adipocyte insulin sensitivity^a^	Systemic insulin sensitivity^b^
**Gene**	**OIR vs OIS**	**IR vs IS**
*CFH*	1.27	1.6
*INPP5A*	0.91	0.67
*KLF15*	0.72	0.63
*LIN7C*	0.93	0.66
*PTP4A1*	0.94	0.66
*SLC2A4*	0.75	0.59
*SLC7A10*	0.74	0.63
*TF*	0.68	0.46
*UBAP2L*	0.94	0.62
*VEGFA*	0.87	0.65
	**OIR vs OIS**	**T2D vs control**^c^
*ACVR1C*	0.81	-21.6
*HADH*	0.87	-17.3
*KIAA1109*	1.09	-6.3
*LOC441666*	1.25	-17.5
*LYRM5*	0.91	-13.1
*NOX4*	1.28	22.6

a. Selected genes differentially expressed in subcutaneous WAT between the OIR and OIS group in the present study (FDR≤5%) (from S1 Table)

b. Elbein et al reported that 172 genes in total were differentially expressed in subcutaneous WAT between insulin resistant and sensitive subjects in analysis of all European and African Americans [9]. Insulin sensitivity was calculated from the insulin-modified, intravenous glucose tolerance test, Individuals in either tail of the distribution of the standardized residual of the insulin sensitivity measurement were categorized as either insulin sensitive (IS) or resitant (IR).

c Nilsson et al reported that 197 genes in total were differentially expressed in subcutaneous WAT between twins discordant for T2D [52].

Limitations of this study include sample size, and the fact that only women were studied. Thus, we cannot from these results draw any conclusions about the control of adipocyte insulin sensitivity in men. Another limitation is that transcriptome profiling on the clinical cohort was performed in WAT specimen whereas the functional studies were limited to adipocytes. Thus, potential indirect effects of stroma-vascular cells on adipocyte insulin sensitivity were not considered in the functional assays.

In conclusion, this study has identified two genes, *KLF15* and *SLC25A10*, potentially important for insulin sensitivity in adipocytes in obese women. *KFL15* and *SLC25A10* inhibit insulin stimulated lipogenesis under conditions when glucose transport is the rate limiting step. In particular *KLF15* could play and important role in development of systemic insulin resistance, as it has also been shown to have effects in other tissues [9, 53].

## Supporting information

S1 Table Genes differentially expressed in subcutaneous adipose tissue between OIR and OIS women with FDR <5%. (XLSX)

S2 Table Gene sets over-represented among differentially expressed genes between OIR and OIS women. (XLSX)

S3 Table Genes differentially expressed between *KLF15* and nontargeting (NEGC) siRNA treated cells with FDR <10%. (XLSX)

S4 Table Correlation with insulin stimulated lipogenes of genes differentially expressed between *KLF15* and nontargeting siRNA treated cells. (XLSX)

S5 Table Genes differentially expressed between *SLC25A10* and nontargeting siRNA (NEGC) treated cells with FDR <10%. (XLSX)

S6 Table Correlation with insulin stimulated lipogenes of genes differentially expressed between SLC25A10 and nontargeting siRNA treated cells. (XLSX)
